# Efficient water scavenging by cooling superhydrophobic surfaces to obtain jumping water droplets from air

**DOI:** 10.1038/s41598-019-50199-9

**Published:** 2019-09-24

**Authors:** Xiaochen Ma, Yang Wang, Heting Wu, Yuanhao Wang, Ya Yang

**Affiliations:** 1 CAS Center for Excellence in Nanoscience, Beijing Key Laboratory of Micro-Nano Energy and Sensor, Chinese Academy of Sciences, Beijing, 100083 P. R. China; 2 Xinjiang Technical Institute of Physics & Chemistry, Chinese Academy of Science, Urumqi, Xinjiang 830011 P. R. China; 3 School of Nanoscience and Technology, University of Chinese Academy of Sciences, Beijing, 100049 P. R. China; 4 Center on Nanoenergy Research, School of Physical Science and Technology, Guangxi University, Naning, Guangxi 530004 P.R. China

**Keywords:** Electronic devices, Devices for energy harvesting

## Abstract

Dew collection is significant in harvesting water and relieving water shortages in arid regions. However, current methods for collecting dew or steam are mainly focusing on the millimeter-sized droplets condensed on the superhydrophobic surfaces. Here, we present a concept for harvesting micro droplets that can spontaneously bounce on the cooling superhydrophobic aluminum surface with randomly micro-nano composite structures, which were fabricated by using a two-step surface structural process. Moreover, an integrated device has been developed, which consists of a triboelectric nanogenerator and the superhydrophobic aluminum sheet. We experimentally explained that the triboelectric nanogenerator, which provides an external electric field by converting wind energy to electric energy with DC voltage pulse peaks of about 60 V, can be utilized to enhance the collection capacity of the jumping water droplets.

## Introduction

With the growth of population, human is suffering water shortages. Condensation is a universal phase-change process and has been one of the most active research fields, due to their promising applications in water harvesting^1–5^. For example, Jiang and co-workers developed multiple biological structures, inspired the cactus with well-distributed clusters of spines, that facilitate efficient fog collection^6^. Wong *et al*. fabricated a slippery rough surface that can be utilized in the water collections^7^. Previous investigations have indicates that surface wettability can affect the ability of water collection^8,9^. Consequently, superhydrophobic surfaces, with low contact angle hysteresis, are regarded as prospective pathway to realize efficient water collection because droplets can easily roll off these surfaces due to gravity upon reaching a critical volume, resulting high condensation efficiency of water^10–15^.

Many efforts have been devoted to explore the features of superhydrophobic surfaces for enhancing condensation heat transfer or efficiency of water harvesting. As a result, droplet jumping, that small condensing droplets bounce from the cooling hydrophobic surfaces because of releasing the superfluous surface energy, has been attracting comprehensive attention. Because the bouncing droplets obtain vitreous electricity, some researchers developed additional electric field to impede bouncing droplets to return back^16,17^. While many studies have focused on structured hydrophobic surfaces to collect droplets from air, methods for harvesting jumping droplets from these surfaces are lacking. Moreover, a great challenge is how to develop feasible technologies by using minimum energy to prevent water droplets to return back. Due to recent advances in harvesting ambient energy as electrical energy, this challenge may be gradually conquered. Triboelectric nanogenerators (TENG) are promising for providing the electric field due to numerous advantages, such as low cost, simple fabrication process, and high output voltages^18–23^.

Here, we demonstrated water harvesting by collecting the jumping droplets based on a superhydrophobic aluminum surface with low temperature. The superhydrophobic surface was fabricated by combining a two-step surface structural process and surface modification with fluoroalkylsilane. It can be found that coalescence-induced droplets on the superhydrophobic surfaces can horizontally jump about 6 mm. Moreover, we used an external electric field, generated by the TENG that can convert ambient wind energy into electric energy, to prevent the droplets to return back to the condensing surface, further enhancing the efficiency of water collection. The rectified TENG with DC voltage pulse peaks of about 60 V can be utilized to enhance the collection capacity of the jumping water droplets. This work could provide insights for the development of new water harvesting technologies from air.

## Results and Discussion

**Figure 1:**
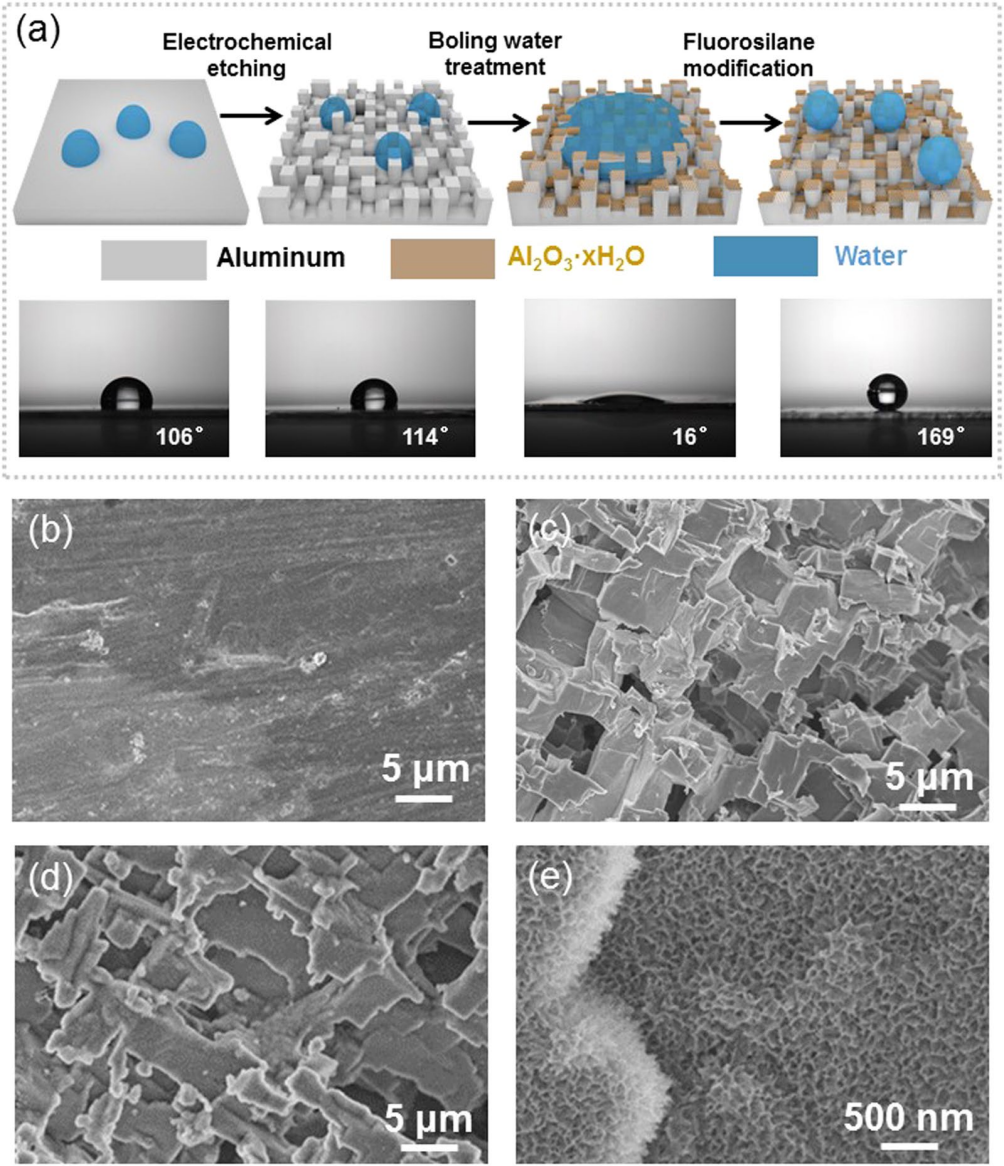
Structure and preparation of the superhydrophobic surfaces. (**a**) Schematic of preparation of the superhydrophobic aluminum surface. (**b**) SEM image of the flat aluminum surface. (**c**) SEM image of the etched aluminum surface. (**d**) SEM image of the boiling water treated surface. (**e**) high-magnification image of the boiling water treated surface.

As aluminium is one kind of the thermal conductive materials that are widely used for devices, we chose aluminium sheets to fabricated superhydrophobic surfaces. Scanning electron microscope (SEM) image of the flat aluminium surface is illustrated in Fig. 1b. To characterize droplet jumping, we firstly fabricated the superhydrophobic aluminium sheets. The process for fabricating the superhydrophobic surfaces comprises chemical etching, boiling water immersion, and fluoroalkylsilane modification (Fig. 1). In the experiment, the aluminium sheet with static contact angle (SCA) of about 106° is first etched in a 2.5 M HCl solution to obtain a micro-structured surface. As displayed in Fig. 1c, irregular micro-protrusions with size of around 5 μm are formed. To achieve hierarchical textures that could enhance droplet departure characteristics, the etched aluminium sheet was immersed in boiling water, resulting in overlying nanostructures on the micro-protrusions (Fig. 1d,e). The formed nanostructures are composed of Al_2_O_3_·xH_2_O, where SCA is about 16°^24^. After fluorosilane modification, the F-containing groups can assemble onto the surface, forming superhydrophobic surfaces. To optimize the superhydrophobicity, we designed a series of surface structures that were controlled by treating time for etching/immersion. Fig. S1 indicates that SCA can reach maximum value of about 169° when etching time is about 5 min and immersion time is about 40 s.

**Figure 2:**
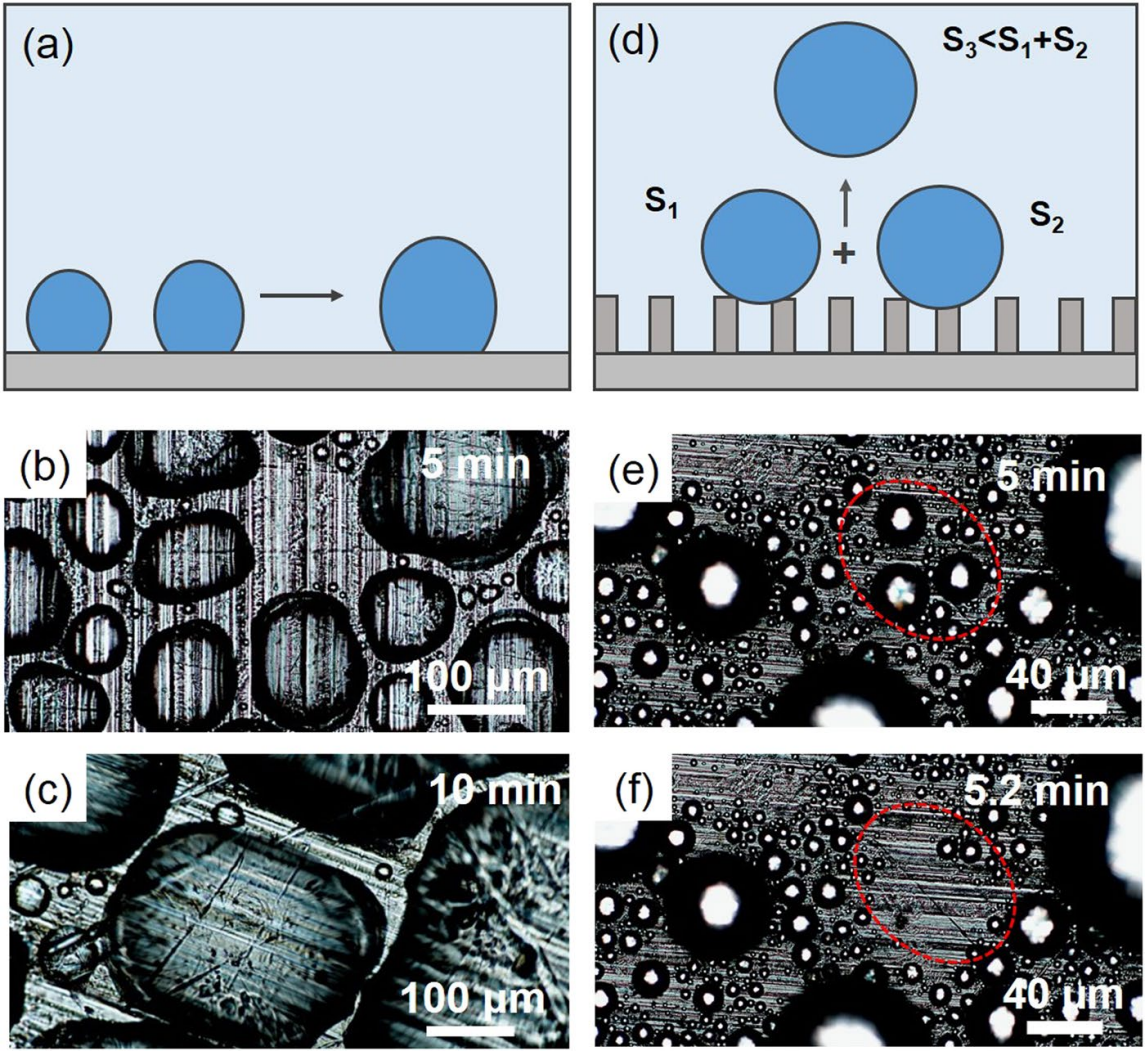
Demonstration of aquatic locomotion for the droplet jumping. (**a**) Schematic diagram of droplet coalescence on the flat surface. (**b**,**c**) Optical images of droplet condensation on the flat surface. (**d**) Schematic of droplet jumping on the superhydrophobic surface. (**e**,**f**) Optical images of jumping droplet condensation on the superhydrophobic surface.

Figure 2a illustrates the schematic diagram of the growth mechanism for droplet on a flat surface. When a pair of small droplets coalesce on a flat aluminium surface with low SCA, they converge each other and no droplets can depart from the surface. As displayed in Fig. 2b,c, small droplets quickly form, and continuously grow on the flat surface fixed on the condensing device at a temperature of −5 °C, where the ambient temperature is about 28 °C (Fig. S2, Supporting Information). Figure 2b illustrates the schematic of the departure mechanism for droplet on the superhydrophobic surfaces. The release of surface energy induced by the merging of small droplets can result in droplet jumping. Figure 2e,f display that some small droplets can jump away from the surface during the condensation experiments.

**Figure 3:**
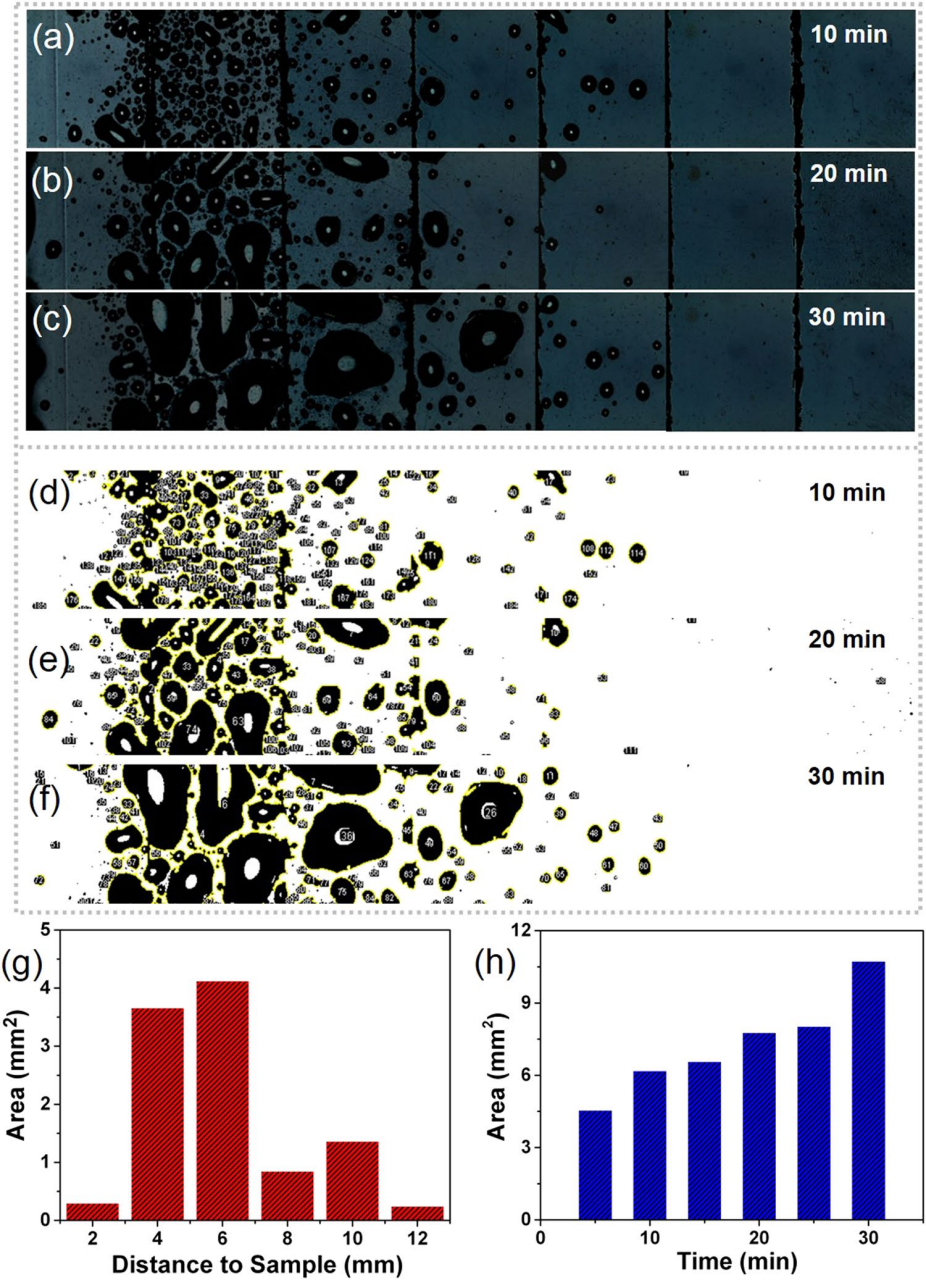
Demonstration of water harvesting. (**a–c**) Optical images of harvesting water from the jumping droplets. (**d–f**) The transformed binary images using threshold algorithm. (**g**) Histogram of experimentally measured harvesting area for the horizontal distance between superhydrophobic aluminum sample and acrylic substrate. (**h**) Histogram of experimentally measured harvesting area at different time.

To characterize droplets jumping away from the surface, we built a custom condensation experimental chamber, where the superhydrophobic sheet vertically was fixed on the condenser, and the acrylic sheet was placed horizontally to collect the jumping droplets (Fig. S3, Supporting Information). The distribution of collected droplets can be achieved by using light microscope. On the basis of a scaling relation between the map size and the sheet, we calculated the jumping distance. The acrylic sheet coated with silver film, treated as a collector for the jumping droplets, was divided into seven parts by graver (Fig. S4, Supporting Information). These parts were observed respectively and then were stitched together as one single image. Figure 3a illustrates that some small droplets appears on the substrates, which are utilized to collect the droplets bounced from the cooling superhydrophobic sheet. With increasing experimental time, the volume of droplets on the substrate enlarge, as displayed in Fig. 3b,c. Moreover, the results indicate that a number of droplets mainly distribute on the partial domain. To offer perspicacity into the experimental results, we use threshold algorithm, which can transform an image to a binary image, to analyse the distribution of droplet jumping (Fig. 3d–f). Figure 3g presents the collected water area as a function of time. Figure 3h displays a histogram of droplet jumping distance measured during the binary image. The distance of droplets travelled away from the superhydrophobic aluminium sheet was between 4 cm and 6 cm. Based on this result, we can optimize the position of water harvesting substrate.

**Figure 4:**
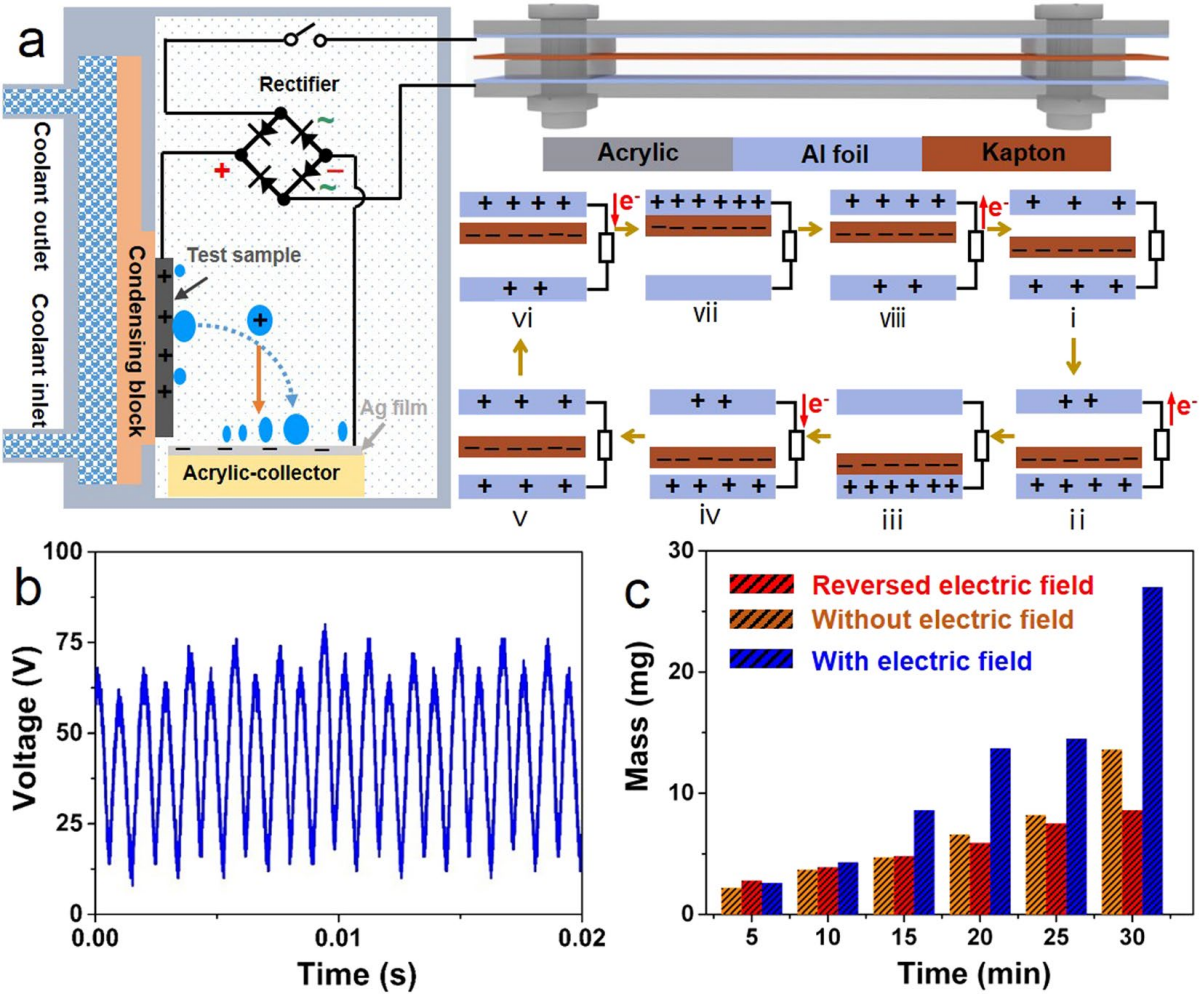
Application of nanogenerator to enhance water harvesting. (**a**) Illustrative diagram of the combined condensation device. (**b**) Measured output voltage signal of the aerodynamics-driven triboelectric nanogenerator. (**c**) Histogram of experimentally measured harvesting water mass as function of time for different electric field.

With previous understanding of enhancement for droplet jumping due to electric-field condensation, we developed a TENG, where an external electric field provided by aerodynamics-driven TENG limits water droplets to return back to the superhydrophobic surface, further enhancing the efficiency of water harvesting^16^. Figure 4a depicts the illustrative diagram of the combined condensation device, where the triboelectric nanogenerator is used to provide additional electrostatic field between the superhydrophobic sample and the acrylic sheet by harvesting wind energy. The vibrating membrane and two Al foils formed a sandwich structure, where the vibrating membrane prepared can quickly vibrate at the windy circumstance, resulting a transform from wind energy to electric energy. The dynamoelectric mechanism is based on the second term of Maxwell’s displacement current^25^. In the original state, no output voltage was generated. During the downward process of the vibration membrane, the generated electrostatic induction can induce electron motion in the opposite direction of vibration of the kapton film. After the separation between the film and the nether electrode, the electrons on the top electrode flow to the starting location. The complete process of the vibrating membrane can result in the AC voltage output in the external circuit. In our experiments, we used an air blower to provide a constant wind speed with about 10 m/s. Then, a rectifier was used to obtain the DC voltage pulse of about 60 V (Fig. 4b). All the water scavenging experiments were implemented in constant temperature and humidity text chamber, which can enable management of relative humidity (RH = 90%).

To investigate the influence of water collection in horizontal voltage field, we consider three conditions that are without electric field, with electric field, and reversed electric field. As illustrated in Fig. 4c, the mass of water collection increases with time, and changes obviously under the electric field. It shows that a 90% higher water mass can be achieved by using the rectified TENG-induced electric field as compared with that without electric field or reversed electric field when the texting time achieves 30 min. These results indicate that the direction of the electric field could affect water collection. When the direction of the electric field is in accord with the jumping droplet, the electric force could limit droplet return and enhance the efficiency of water collection due to electrostatic force. On the contrary, the jumping droplet could be restrained. The main reason is that jumping droplets can obtain a net positive charge. In general, superhydrophobic surfaces could obtain OH^─^ from the nucleated droplet. When the droplet quickly jumps from the surface, it usually holds H^+^^26^. However, this phenomenon disappears due to form a counter electric field when the droplet is removed slowly. In this work, we fabricated an aerodynamics-driven generator (100 × 10 × 2 mm^3^), which engendered an output voltage of 60 V. Previous studies indicate that the intensity of electric field can affect the droplet jumping^16,17^. Different output voltages can be easily realized by changing the size of the TENG, where some TENGs can generate the output voltage of about 300 V^27^.

Based on our results, we present an effective method to promote droplet jumping on the superhydrophobic surfaces based on an aerodynamics-driven TENG. It is well known that the high output voltage is one of main features of TENGs. Thus, we use the aerodynamics-driven TENG to establish electric field, which can effectively limit the jumping droplets return. Due to ubiquitous wind energy, aerodynamics-driven TENGs can be used to enhance droplet jumping.

## Conclusion

In conclusion, we have demonstrated the concept for collecting jumping water droplets on the cooling superhydrophobic aluminum surfaces with a contact angle of about 169°. Most of the collecting water droplets have a distance of about 6 mm with the vertical superhydrophobic surfaces. Moreover, a wind-driven rectified TENG with DC voltage pulse peaks of about 60 V has been utilized to enhance the collection capacity of the jumping water droplets, where a 90% mass enhancement can be confirmed as compared with that of without using of TENG in 30 min collecting process. The fundamental concepts exhibited in this study could also give a guideline to enhance water collection by using ambient energy in regions that are suffering water shortages.

## Methods

### Fabrication of superhydrophobic aluminum surface

Al sheets, with the dimensions of 10 × 10 mm^2^, were ultrasonically cleaned ethanol for 10 min. Subsequently, the sheets were washed by deionized water, and dried at 90 °C. The samples were immersed in an etching solution (16 mL HCl and 84 mL H_2_O) for different time to fabricate micro-structured surface. Then etched plates were ultrasonically cleaned, and dried at 90 °C for 30 min. Subsequently, nanostructures on the surface were fabricated by the boiling water immersion for 40 s. The plates were dried at 90 °C in vacuum drying oven. Then the plates were immersed in a 1.0 wt % toluene of 1 H,1 H,2 H,2H-perfluorooctyltriethoxysilane for 30 min, and then dried at 100 °C for 60 min.

### Fabrication of TENG and silver film

By the laser processing, the acrylic plates with two holes were made into substrates (100 × 10 × 5 mm^3^). Two Al foils were stuck on the substrates. The kapton film was fixed between two acrylic substrates by using supporting beams and can contact Al foil periodically due to wind-induced vibration. Silver film was deposited on the surface of acrylic sheet by DC magnetron sputtering with a power of 100 W.

### Characterization and measurement

The surface structures of the prepared aluminum surface were determined by field emission scanning electron microscopy (FESEM, SU8020, Hitachi). Water contact angles of the prepared superhydrophobic surfaces were obtained by a contact angel meter (SC1300F, China) at room temperature. The droplet jumping was observed by using an optical microscopy (NIKON, LV100ND). The output voltage signals were measured by a digital phosphor oscilloscope (Tektronix MDO 3024).

## Supplementary information

Supplementary Information
