# Supplementary material for: Efficient water scavenging by cooling superhydrophobic surfaces to obtain jumping water droplets from air
Source: Sci Rep. 2019 Sep 24;9:13784. doi: 10.1038/s41598-019-50199-9 (PMC6760228; doi:10.1038/s41598-019-50199-9)
Supplement: Supplementary file 1 — Supplementary Information [file 41598_2019_50199_MOESM1_ESM.docx]

Supplementary for

Efficient water scavenging by cooling superhydrophobic surfaces to obtain jumping water droplets from air

*Xiaochen Ma,^#1^ Yang Wang,^#13^ Heting Wu,^13^ Yuanhao Wang^*3^ and Ya Yang^*134^*

**
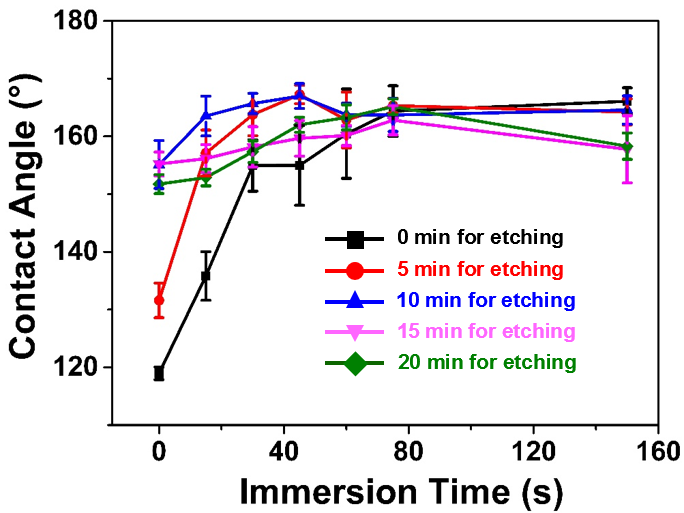
**

**Fig. S1 Relationship between the reaction time and contact angle for different etching time.**


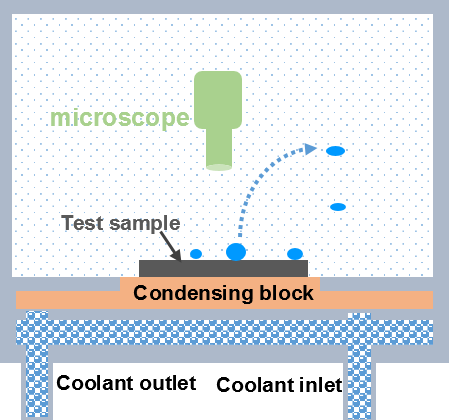


**Fig. S2 Schematic diagram of the condensation device.**

**
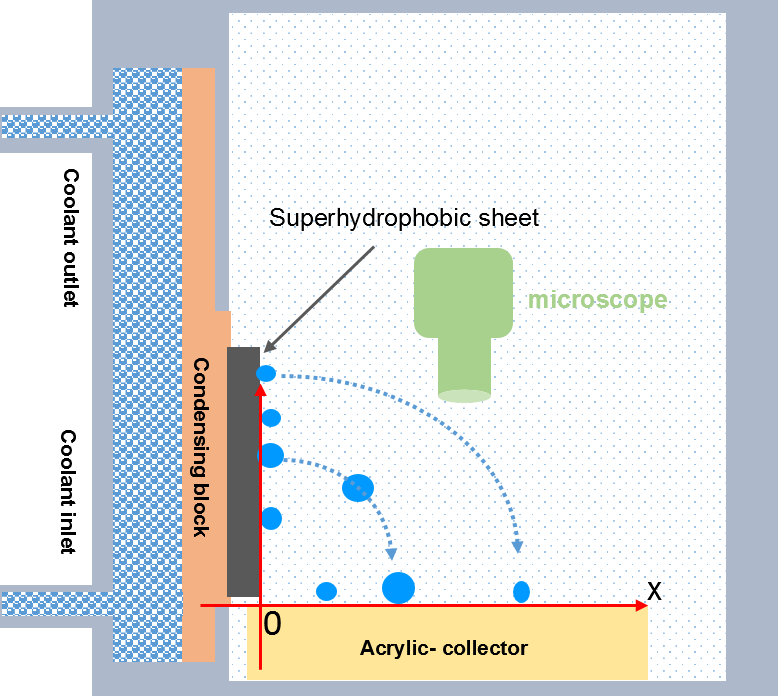
**

**Fig. S3 Schematic diagram of the condensation device for studying jumping distance of droplets.**


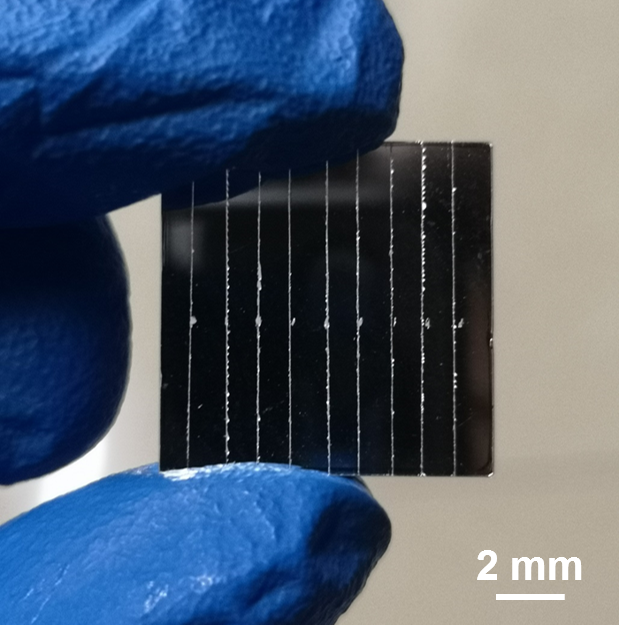


**Fig. S4 The photography of** **acrylic sheet.**
